# The Role of Short-Form Video Apps in Mitigating Occupational Burnout and Enhancing Life Satisfaction Among Healthcare Workers: A Serial Multiple Mediation Model

**DOI:** 10.3390/healthcare13040355

**Published:** 2025-02-07

**Authors:** Donghwa Chung, Yanfang Meng, Jiaqi Wang

**Affiliations:** 1 School of Journalism and Communication, Central China Normal University, Wuhan 430079, China; chungdonghwa@ccnu.edu.cn (D.C.); jiaqiwang@mails.ccnu.edu.cn (J.W.); 2 School of Journalism and Communication, Beijing Institute of Graphic Communication, Beijing 102699, China

**Keywords:** occupational burnout, healthcare workers, short-form video apps, life satisfaction, survey

## Abstract

**Background**: The intersection of occupational burnout and digital leisure activities has garnered increasing scholarly attention in recent years. However, limited research has examined how Chinese healthcare workers engage with short-form video apps as a stress management tool. **Objectives**: This study employs a serial multiple mediation model to explore the impact of occupational burnout on the use of short-form video apps and its subsequent effects on a sense of community, intrinsic rewards, and life satisfaction among Chinese healthcare workers aged 18–34. **Methods**: Data were collected through an online survey, with 362 valid responses, and analyzed using descriptive statistics, hierarchical regression, and mediation analyses, including serial mediation via SPSS 25.0. **Results**: The results demonstrate a positive direct association between occupational burnout and the use of short-form video apps. Furthermore, the relationship between the use of short-form video apps and life satisfaction was mediated through two distinct pathways, namely, a sense of community and intrinsic rewards. **Conclusions**: These findings contribute to the expanding body of literature on the role of digital media in stress management and well-being among healthcare workers, as well as highlighting evidence-based digital interventions to support healthcare workers’ well-being in high-stress settings.

## 1. Introduction

Occupational burnout is widely acknowledged as a major outcome of occupational stress, impacting employees across diverse sectors [1]. It is defined as a syndrome caused by prolonged workplace stress and characterized by several consequences, such as feelings of detachment, reduced professional efficacy, and fatigue, which negatively impact employees’ well-being and health [2]. Burnout among healthcare workers is a growing global concern, with studies reporting high prevalence rates across various countries. Research in Iran, the Republic of Korea, and the Czech Republic has indicated significant levels of emotional exhaustion, depersonalization, and disengagement among healthcare professionals [3,4,5]. For example, a cross-sectional survey in the Czech Republic found that 46.24% of healthcare workers reported high emotional exhaustion, 25.56% experienced significant depersonalization, and 24.15% reported low levels of personal accomplishment.

Healthcare workers are highly susceptible to burnout due to the demanding nature of their roles. These professionals frequently manage emergency cases [6], endure long shifts [7], maintain an intense focus on patient care [8], and experience sleep deprivation [9]. Studies have reported that these challenges result in limited opportunities for rest, both during and between shifts, thereby hindering adequate recovery [10]. Over time, persistent stressors contribute to occupational burnout, adversely affecting healthcare workers’ job performance, work–life balance, and overall life satisfaction [11,12].

In recent years, occupational burnout among healthcare workers has been steadily increasing worldwide, emphasizing the critical need to identify contributing factors and implement effective strategies to mitigate psychological stress [13]. Extensive research has highlighted that healthcare workers across various countries benefit from engaging in recreational activities, leisure pursuits, and physical exercise, with these interventions being positively associated with greater life satisfaction [14,15]. Furthermore, a descriptive study underscored that promoting participation in leisure activities can significantly enhance both the physical and mental well-being of healthcare workers [15]. While the relationship between occupational burnout, leisure activities, and life satisfaction has been extensively examined, emerging evidence suggests that digital leisure engagement represents a promising global intervention for alleviating burnout and enhancing the overall well-being of frontline healthcare workers.

Digital leisure activities encompass a variety of entertainment, socialization, and communication forms facilitated by digital tools or platforms [16]. These activities not only offer relaxation but also provide brief breaks from work, contributing to enhanced mental resilience and improved energy levels [17]. As digital platforms evolve and new media forms emerge, short-form videos have become a dominant medium, driving significant user engagement worldwide [18]. Among these platforms, Douyin, the Chinese counterpart of TikTok, has gained immense popularity not only in Western countries but also across East Asia, particularly in China. According to the 2024 Douyin User Data Report, the platform boasts over 250 million daily active users in China [19]. Additionally, Douyin generates more than 3 million daily content uploads, underscoring its extensive influence and reach [20]. Short-form video apps have firmly established themselves as critical channels for accessing information and facilitating social interaction within the Chinese context. Douyin’s seamless integration into daily routines has transformed it into a cultural norm, solidifying its role as an essential part of life for millions of Chinese citizens.

The impact of social media use on psychological health has been extensively examined, with research highlighting both positive and negative effects [21,22,23,24]. However, growing evidence suggests that platforms such as Facebook, Twitter, and TikTok can contribute to life satisfaction [25,26]. While prior studies have significantly advanced the understanding of the relationship between social networking site use and life satisfaction across various occupations, such as cruise ship employees, digital strategists, and fintech marketers [27,28], research on critical professions, particularly healthcare workers, remains limited. A review of the relevant literature reveals two notable research gaps. First, limited studies have explored whether healthcare workers benefit from emerging media, such as short-form video apps, during brief leisure periods and how these platforms may contribute to mental resilience and energy restoration. Second, the mechanisms underlying the formation of life satisfaction resulting from the use of such platforms remain largely unexplored, with empirical studies yet to investigate these processes in depth. Therefore, the present study aims to investigate the impact of the use of short-form video apps (USFVA) among Chinese healthcare workers, referring to their engagement with various short-form video platforms for content creation, uploading, and interaction. This engagement includes a range of activities, such as accessing entertainment, educational resources, and industry-related content, as well as participating in in-app interactions, including liking, commenting, sharing, and socializing with other users.

As highlighted above, this study, grounded in the theoretical framework of sense of community, investigates a serial multiple mediation model. Specifically, it examines how occupational burnout influences the USFVA, which subsequently enhances (1) a sense of community, leading to improved life satisfaction, and (2) intrinsic rewards, which also contribute to improved life satisfaction among Chinese healthcare workers aged 18–34 years old. The findings provide a nuanced understanding of the complex relationship between occupational burnout and life satisfaction, offering practical guidance for addressing these challenges in healthcare settings.

## 2. Literature Review

### 2.1. Theoretical Framework of Sense of Community

In the span of recent decades, critical and influential theories have contributed to understanding the psychological and cognitive processes underlying how individuals interact within groups and communities. For instance, Maslow’s Hierarchy of Needs (1943) [29], Tajfel’s Social Identity Theory (1979) [30], and Deci and Ryan’s Self-Determination Theory (1985) offer crucial insights into how individuals derive meaning, motivation, and connection from their social environments [31]. While these theories provide valuable perspectives on how key components of personal growth and intrinsic motivation foster individuals’ sense of identity, they often fail to offer an in-depth explanation of the development and dynamics between individuals and their communities, particularly from a wider societal or group perspective. In response to this limitation, McMillan and Chavis (1986) proposed the sense of community theoretical framework to address the explanatory gap [32]. A sense of community refers to a feeling of belonging, mutual importance, and a shared belief in fulfilling members’ needs through group commitment. The concept consists of four key elements: membership, influence, integration, and fulfillment of needs (McMillan & Chavis, 1986) [32]. In the context of the current study, it is defined as a sense of belonging, shared aspirations, and a strong commitment to meeting the needs of Chinese healthcare workers who use short-form video apps. Prior studies have shown that this theoretical framework has been applied across various fields of study, particularly in understanding how the sense of community plays a crucial mediating role in the relationship between the use of online platforms and individuals’ well-being. This body of research has contributed to our understanding of how a feeling of belonging and shared connection on online or media platforms can reinforce perceived life fulfillment, foster improved mental health, and encourage engagement in self-care practices [33,34,35].

As discussed above, there are two key reasons why this theory is well suited to guide this study. First, prior research demonstrates the strong applicability and explanatory power of the sense of community framework in media community platforms. Specifically, the framework has proven robust in understanding both the antecedents and outcomes of phenomena within social media environments [36,37]. Second, considering the collective dimensions of media communities, particularly in Chinese short-form video apps, this theory provides a lens through which to gain an in-depth understanding of how users’ psychological rewards are met within these app communities.

### 2.2. Douyin’s Emerging Role as a Platform for Healthcare Workers in China

Short-form video apps such as Douyin and Kuaishou have become some of the most dominant digital platforms. For instance, Douyin stands out as a leading platform, with approximately 250 million daily active users in China [19]. The app provides content that meets a diverse range of informational needs, with over 3 million content uploads generated daily. One noteworthy recent development is the growing visibility of healthcare professionals on short-form video apps, which has garnered considerable attention. As of March 2023, it was reported that over 35,000 certified doctors from leading hospitals had joined the platform [38]. Furthermore, data on daily usage indicate that over 30% of nurses spend more than two hours on such platforms each day, while nearly one-quarter use the platform for one to two hours [39]. By sharing information through their content, healthcare professionals are actively engaged in disseminating knowledge related to health education, rehabilitation, and disease prevention.

In light of the aforementioned observation, the existing literature elucidates the motivations of healthcare workers from a user gratification perspective. Beyond the general USFVA for social and educational purposes, there is a pressing need to relieve job-related stress. Stress has emerged as a significant motivator for healthcare workers’ engagement with short-form video apps. By addressing their social, educational, and entertainment needs, such platforms have the potential to alleviate work-related stress, promote mental resilience, and enhance energy levels, all of which are closely tied to overall well-being and user satisfaction. However, despite this potential, research has yet to explore the mediated mechanisms that link occupational burnout to life satisfaction through the USFVA.

In addition, this study exclusively focuses on healthcare workers aged 18–34 years for two primary reasons. First, this age group has been identified as particularly vulnerable to occupational burnout due to longer shifts and higher job demands compared to older healthcare workers [40]. Second, as members of Generation Z and Millennials, they have grown up in a highly digitalized environment [41]. As a result, they engage more frequently with emerging digital platforms, particularly short-form video apps. Their high level of digital engagement indicates a greater likelihood of utilizing these platforms for stress relief compared to older age groups. Examining these mechanisms can provide valuable insights into practical strategies for mitigating burnout and enhancing life satisfaction among healthcare workers.

### 2.3. The Effect of Occupational Burnout on USFVA

Due to the fast-paced nature of various work environments, heavy workloads, and limited time between shifts, workers often face restricted opportunities for rest, leading to a heightened sense of occupational burnout [6,7,8]. Prior studies have demonstrated that engaging in digital leisure has become a popular stress-coping strategy for individuals experiencing occupational burnout [42]. For instance, research has shown that individuals with higher levels of emotional exhaustion and stress (commonly referred to as burnout) tend to use social media more frequently [43]. This suggests that during periods of burnout, individuals are more likely to turn to social media as a coping mechanism to mitigate exhaustion and alleviate stress.

Similarly, an empirical study demonstrated that engagement in online leisure activities significantly increases in response to psychological distress (commonly referred to as burnout) [44]. In other words, individuals experiencing higher levels of occupational burnout tend to engage in online leisure activities, such as browsing websites, more frequently than those with lower levels of psychological distress. Furthermore, a three-wave longitudinal study confirmed that individuals are more likely to use the internet as their levels of occupational burnout increase [45].

The above-mentioned scientific findings indicate that occupational burnout may influence healthcare workers’ USFVA. Therefore, the following hypothesis is proposed:

**Hypothesis 1.**  *Occupational burnout has a significant and positive effect on healthcare workers’ USFVA.*

### 2.4. The Mediating Role of Sense of Community

A nuanced psychological perspective suggests that social media use may foster users’ external connections with others, such as maintaining relationships, strengthening relational bonds, and perceiving emotional support [46]. These effects collectively enhance users’ need for a sense of community. As this factor has garnered significant attention from scholars across various fields, prior studies have identified the mediating role of a sense of community in the relationship between social media use and life satisfaction. For instance, frequent Facebook users tend to exhibit higher levels of a sense of community [47]. Additionally, research has found a positive association between a sense of community and life satisfaction [48]. In line with this research, previous studies have confirmed that social media plays a pivotal role in promoting social connectedness, also known as a sense of community [49]. Furthermore, Hombrados-Mendieta et al. (2019) demonstrated that individuals with a stronger sense of community tend to experience higher life satisfaction [50]. Based on a review of these scientific and empirical studies, this study posits that a sense of community may indirectly influence the relationship between USFVA and life satisfaction among Chinese healthcare workers. Therefore, the following hypothesis is proposed:

**Hypothesis 2.**  *Sense of community mediates the relationship between USFVA and Chinese healthcare workers’ life satisfaction.*

### 2.5. The Mediating Role of Intrinsic Rewards

Reward is considered a psychological process in which the brain associates certain actions or experiences with positive outcomes, leading individuals to experience emotional reactions and feelings of satisfaction. While the investigation of rewards has attracted considerable attention from psychologists and behavioral scholars, a line of research argues that different types of rewards exert distinct influences on specific needs and fulfill various types of personal satisfaction. Broadly, rewards can be categorized into two major types: extrinsic and intrinsic [51]. Extrinsic reward is defined as outward benefits, often seen as tangible or visible bonuses, that motivate individuals to complete tasks or achieve goals [52]. Such rewards are driven by external factors rather than inner satisfaction or enjoyment. For instance, examples of extrinsic rewards include receiving bonuses, promotions at work, and recognition [53]. Intrinsic reward, on the other hand, is defined as the inherent satisfaction individuals derive from completing tasks that are perceived as worthwhile or beneficial. Examples include personal growth, self-development, and the pursuit of knowledge, which are considered intrinsic rewards [54]. In the current study, intrinsic rewards refer to the inner gratification and joy individuals experience through personal growth, self-development, and the exploration of knowledge when using short-form video apps.

While the impact of both extrinsic and intrinsic rewards has been explored, empirical studies have confirmed that intrinsic rewards stand out as significant enhancing factors that indirectly improve individuals’ life satisfaction through engagement in leisure activities. This can be explained by the fact that extrinsic rewards often reduce individuals’ intrinsic task motivation, creativity, and satisfaction [55]. Such rewards are typically short-term and fail to generate sustained life satisfaction [56]. In contrast, intrinsic rewards are more grounded in personal fulfillment and inner gratification, which contribute to longer-lasting life satisfaction [57,58]. Employees who receive higher levels of intrinsic rewards tend to exhibit greater autonomy and actively seek opportunities for professional growth, all of which contribute to sustained life satisfaction [57]. Moreover, individuals who prioritize intrinsic rewards often demonstrate a deep conviction in the value of inner growth and personal gratification. This focus helps them build resilience against the fleeting influence of external rewards, such as bonuses or public recognition. As mentioned earlier, Chinese short-form video apps provide entertainment and educational content aimed at knowledge growth. It is essential to further investigate how engaging in these emerging modern forms of recreation, such as the USFVA, enhances intrinsic rewards and, ultimately, contributes to improved life satisfaction.

Prior empirical studies have indicated that social network use enhances individuals’ perceived joy in learning (intrinsic rewards) [59]. Moreover, intrinsic rewards have been positively associated with well-being [60]. Additionally, social media use and emotional gratification were found to be significantly positively associated [61]. In other words, individuals who frequently use social media tend to reinforce their levels of intrinsic rewards. Furthermore, Wiedemann et al. (2014) found that higher levels of intrinsic rewards are associated with greater life satisfaction [62]. After critically reviewing the empirical literature, the current study proposes that intrinsic rewards serve as a critical mediating mechanism in the relationship between USFVA and life satisfaction among Chinese healthcare workers. Thus, the following hypothesis is proposed:

**Hypothesis 3.**  *Intrinsic rewards mediate the relationship between USFVA and Chinese healthcare workers’ life satisfaction.*

### 2.6. The Serial Mediating Role of USFVA and Psychological Factors

Previous research has explored the impact of social media use on life satisfaction through external connections (a sense of community) and internal motivations (intrinsic rewards). However, critical empirical studies suggest that there may be nuanced and potentially serial relationships between occupational burnout and life satisfaction. For example, a positive association has been observed between occupational burnout and social media use [43]. Moreover, frequent Facebook users tend to report higher levels of a sense of community [47], which, in turn, leads to greater levels of life satisfaction [48].

Aligned with such studies, recent research has suggested that social media use and intrinsic rewards may serve as serial mediators in the relationship between occupational burnout and life satisfaction among Chinese healthcare workers. For instance, a previous study noted that psychological distress encourages individuals to engage in online leisure activities, such as social media use [44]. Furthermore, empirical evidence has demonstrated a significant positive association between social network use and individuals’ perceived joy in learning (intrinsic rewards) [59]. Additionally, intrinsic rewards have been shown to enhance individuals’ life satisfaction [62]. These findings collectively provide valuable evidence supporting the role of a sense of community and intrinsic rewards as serial mediators. We, thus, propose the following serial mediation mechanism in the relationship between occupational burnout and life satisfaction: USFVA → a sense of community, USFVA → intrinsic rewards. Therefore, the following hypotheses are proposed:

**Hypothesis 4.**  *USFVA and a sense of community play a serial mediating role in the relationship between occupational burnout and Chinese healthcare workers’ life satisfaction.*

**Hypothesis 5.**  *USFVA and intrinsic rewards play a serial mediating role in the relationship between occupational burnout and Chinese healthcare workers’ life satisfaction.*

**Figure 1:**
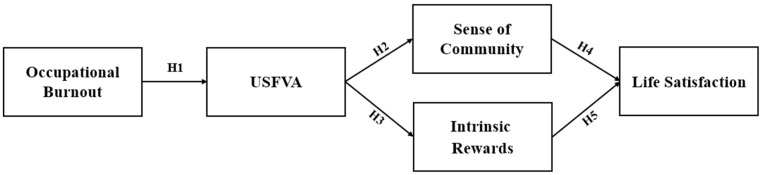
A hypothesized model illustrating the impact on healthcare workers’ life satisfaction.

In summary, Figure 1 demonstrates the hypothesized model’s influence on Chinese healthcare workers’ life satisfaction.

## 3. Methods

### 3.1. Data Collection

Following He, Z. and Chen, M. (2024), an online pilot survey was conducted in October 2024 to assess the accuracy and consistency (validity and reliability) of all measures [63]. The survey included 45 healthcare professionals (medical interns, physicians, surgeons, and pharmacists) and 40 healthcare support workers (nurses and aides) recruited through Tencent Questionnaires (https://wj.qq.com/ accessed on 9 October 2024), one of the most widely used online survey platforms in China. The pilot results indicated acceptable reliability (α ≤ 0.86) and strong construct validity, supported by significant Kaiser–Meyer–Olkin (KMO) and Bartlett’s test results. Additionally, all factor loadings exceeded 0.5, confirming robust measurement qualities [64].

The official online survey was conducted in October 2024 using the same online questionnaire platform. With a database of over 48 million Chinese individuals authenticated by real name [65], the platform facilitated the recruitment of healthcare professionals from across provinces and cities in China. The study sample was randomly selected from the pool provided by the survey company. Invited participants received a link to the online questionnaire, which included an informed consent form on the first page. This form outlined the purpose and procedures of the study and informed participants of their right to discontinue participation or withdraw their consent at any time. Additionally, it assured them of the strict confidentiality of all information provided [66,67].

**Figure 2:**
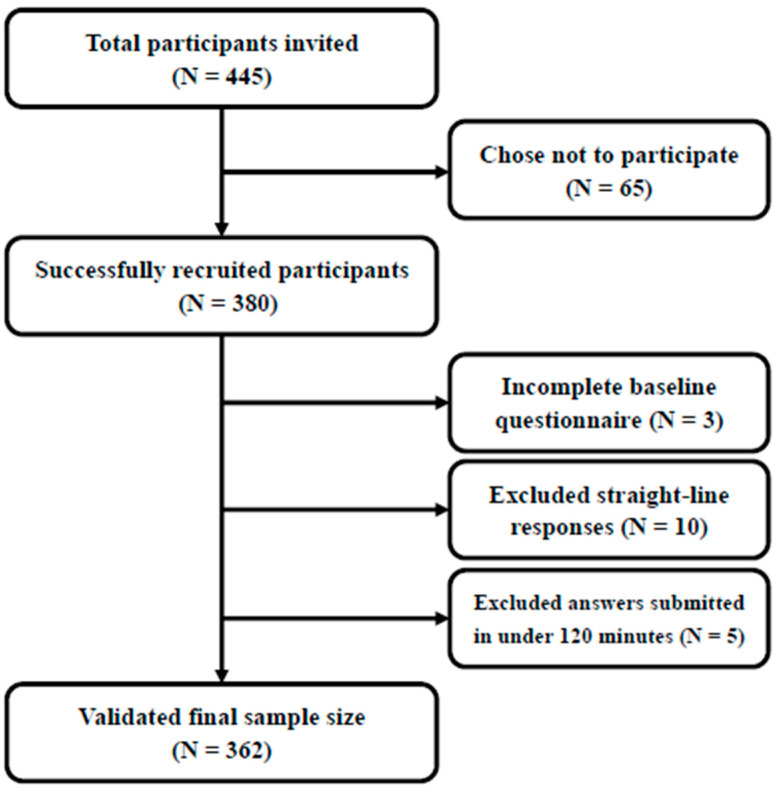
Flow chart of the respondent enrollment procedure.

The eligibility criteria for this study were as follows: (1) current employment as a healthcare worker (e.g., doctors, nurses, aides, helpers, and other professionals), (2) aged between 18 and 36 years, and (3) prior use of short-video apps such as Douyin, Kuaishou, or WeChat Channels. Participants were invited to complete the survey between 10 October 2024 and 10 December 2024. Ethical approval for this study was granted by the Academic Committee of the School of Journalism and Communication, Beijing Institute of Graphic Communication (BIGC20241008, 8 October 2024). Over 445 healthcare professionals were invited to complete the online questionnaire, with a total of 380 respondents participating. To ensure data quality, three data-cleaning methods adopted from previous studies were implemented. The detailed enrollment procedure is illustrated in Figure 2. The final valid sample comprised 362 responses.

Previous research has employed G-Power 3.1 to estimate the minimum required sample size, a method widely used in empirical studies [68]. In the current study, the analysis was conducted using the following settings: an effect size of 0.15, a power of 0.80, and four predictors. Based on these settings, the calculated minimum sample size required to achieve statistical significance was 85. Additionally, a Monte Carlo power test was performed to determine the minimum sample size required for two partial mediation models. This method, widely accepted in psychological studies [69,70], included 500 replications, a sample size step of 10, and a significance level of 0.05. The results indicated that, with a power of 0.80, the minimum required sample size was 230. The sample size in the current study (N = 340) exceeded these calculated minimum requirements, confirming its adequacy for the intended analyses. Therefore, the sample is considered sufficient.

### 3.2. Measurements

The questionnaire items were carefully reviewed and developed, drawing on prior studies [71,72,73,74,75,76,77,78]. All items used in this study were measured on a 5-point Likert scale, ranging from 1 (never) to 5 (most frequently) or 1 (strongly disagree) to 5 (strongly agree). The selection of a 5-point Likert scale is justified by its ease of administration, scoring, and comprehension. Furthermore, this measurement approach is widely recognized as an effective method for deriving statistical inferences with enhanced accuracy and reliability [79]. Among the measurements, intrinsic rewards capture an individual’s inner gratification and joy. To our knowledge, no prior study has developed an intrinsic reward measurement specifically in the context of China or with a focus on Chinese healthcare workers as a demographic.

To develop a concise and accurate measure of Chinese healthcare workers’ intrinsic rewards, the processes outlined by Rehman (2023) and Manzoor et al. (2021) guided this study [80,81]. First, three experts in health communication were invited to participate. These experts were selected from the Beijing Institute of Graphic Communication and Central China Normal University. Second, the measure was carefully refined, focusing on four items using a five-point Likert scale with options labeled “clear” and “not clear”. Based on the experts’ evaluations and suggested revisions, the measure underwent further modifications.

To ensure a high standard of accuracy and reliability, focus groups were conducted with 10 volunteers, and content and face validity methods were applied before the pilot study involving a sample of 95 Chinese healthcare workers. These methods specifically addressed and resolved any ambiguous or unclear items [82]. The final measurements, including each item’s factor loadings, composite reliability (CR), and average variance extracted (AVE), are presented in Appendix A.

Numerous cross-sectional survey studies emphasize the importance of addressing common method bias (CMB) to enhance convergent validity and minimize potential statistical errors [22]. In this study, Harman’s single-factor analysis was conducted, a method commonly used in psychiatric research [83]. The analysis revealed that a single factor accounted for 34.88% of the variance, well below the recommended threshold of 50%. Thus, CMB was not identified as a significant concern in this study.

The Fornell–Larcker criterion, validated in previous research as an effective method for assessing discriminant validity [84], was applied to confirm that each measurement is distinct and captures a unique construct. As shown in Table 1, each average variance extracted (AVE) score exceeded the corresponding correlations with other factors. Therefore, the measurements were confirmed to be distinct.

### 3.3. Data Analysis Methods

This study collected 362 valid responses through an online questionnaire from 10 October to 10 December 2024. Data analysis was performed using SPSS 25.0. The analysis steps were as follows: (1) reliability and validity tests, (2) descriptive statistics (including age, gender, and education), (3) hierarchical regression analysis to assess direct effects, and (4) the PROCESS macro (Models 4 and 6) to test both mediated and serially mediated hypotheses. To enhance the accuracy of estimates and assess the significance of the mediation paths, bootstrapping was applied to generate bias-corrected 95% confidence intervals.

## 4. Results

### 4.1. Descriptive Data

In total, 362 valid samples were collected. Table 2 presents the key demographic characteristics of the respondents. The majority of the healthcare workers were male (N = 197, 54.4%). Most respondents had undergraduate (N = 285, 78.7%) or postgraduate (N = 41, 11.3%) education. Additionally, the majority were between 18 and 23 years old (N = 202, 55.8%), followed by those aged 30 to 34 years and older (N = 97, 26.8%) and then 24 to 29 years old (N = 63, 17.4%). Lastly, regarding monthly income, most respondents earned between CNY 8001 and 15,000 (N = 119, 32.9%) or between CNY 5001 and 8000 (N = 89, 24.6%). Lastly, most healthcare workers were medical interns (N = 201, 55.5%) or nurses (N = 50, 13.8%), followed by physicians and surgeons (N = 41, 11.3%).

### 4.2. Hypothesis Testing

To test Hypothesis 1, hierarchical regression analysis was conducted using SPSS 25.0. In the first block, demographic factors, including education and income, were included as confounding variables to control for their potential influence on the dependent variable [85]. This approach ensured that the observed effects were attributable to the independent variable rather than demographic differences. In the second block, occupational burnout was entered as the independent variable. Finally, USFVA was entered as the dependent variable. The results showed that the first regression model was statistically significant, explaining 11% of the variance (R^2^ = 0.11, *p* < 0.05). Moreover, the direct effect of occupational burnout on the USFVA was significant (β = 0.13, t = 2.00, *p* < 0.05). Therefore, Hypothesis 1 is supported.

To test the mediated hypotheses of the current study (Hypotheses 2 and 3), Hayes’ PROCESS macro model 4 was exclusively used. Following the analysis process of prior studies, bootstrapping was used to obtain bias-corrected 95% confidence intervals, ensuring the accuracy of estimates and assessing the significance of the mediation models [86]. In the first mediation model, the USFVA was positively associated with a sense of community (β = 0.34, t = 9.16, *p* < 0.001). A sense of community had a positive effect on life satisfaction (β = 0.31, t = 6.47, *p* < 0.001). Furthermore, the indirect effect was significant (β = 0.11, t = 9.19, *p* < 0.001, 95% CI [0.06, 0.16]), confirming the mediating effect of a sense of community. In the second mediation model, the USFVA was positively associated with intrinsic rewards (β = 0.16, t = 5.03, *p* < 0.001). Intrinsic rewards had a positive effect on life satisfaction (β = 0.34, t = 5.92, *p* < 0.001). Additionally, the indirect effect was significant (β = 0.10, t = 9.20, *p* < 0.001, 95% CI [0.03, 0.10]), providing robust evidence for the mediating role of intrinsic rewards. In conclusion, these findings fully support Hypotheses 2 and 3.

**Figure 3:**
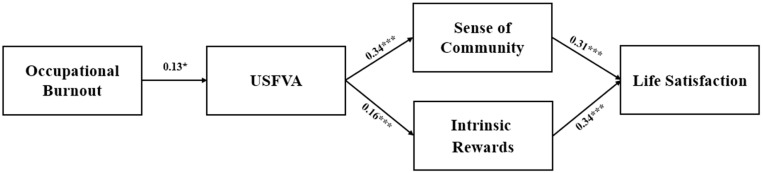
A model of predictors for healthcare workers’ life satisfaction. * *p* < 0.05, *** *p* < 0.001.

Finally, Hypotheses 4 and 5 were tested using Hayes’ PROCESS macro model 6, which has been widely used in previous studies to investigate serial mediation effects. The results for the first serial mediation model indicated that occupational burnout had a positive effect on the USFVA (β = 0.13, t = 2.00, *p* < 0.05). The USFVA had a positive effect on a sense of community (β = 0.33, t = 8.90, *p* < 0.001) and life satisfaction (β = 0.23, t = 6.15, *p* < 0.001). In addition, a sense of community positively predicted life satisfaction (β = 0.34, t = 7.12, *p* < 0.001), but the serial mediation effect was not significant (β = 0.01, t = −1.54, *p* > 0.05, 95% CI [−0.01, 0.04]). Hypothesis 4 was, therefore, rejected. The results of the second serial mediation model indicated that occupational burnout had a positive effect on the USFVA (β = 0.13, t = 2.00, *p* < 0.05). The USFVA had a positive effect on intrinsic rewards (β = 0.15, t = 5.00, *p* < 0.001) and life satisfaction (β = 0.30, t = 8.14, *p* < 0.001). In addition, intrinsic rewards positively predicted life satisfaction (β = 0.37, t = 6.520, *p* < 0.001), but the serial mediation effect was not significant (β = 0.01, t = −1.54, *p* > 0.05, 95% CI [−0.01, 0.02]). Hypothesis 5 was, hence, rejected. Table 3 provides a summary of the hypothesis testing results. In addition, Figure 3 presents the standardized coefficients and significance levels for each effect (direct, mediated, and serially mediated) within the hypothesized model.

## 5. Discussion

Occupational burnout among healthcare workers is strongly linked to facing emergency cases, enduring long shifts, and maintaining an intense focus on patient care. Accumulated burnout, in particular, contributes to negative stressors that affect job performance and life satisfaction. Given this, it is essential to investigate whether Chinese healthcare workers can benefit from emerging media, with brief periods of relaxation during leisure time potentially enhancing their life satisfaction. Furthermore, the current study aligns with prior literature examining the impact of social media use on life satisfaction across various occupations [27,87], extending this research to frontline workers, particularly healthcare professionals. Guided by the theoretical framework of sense of community, this study examined a serial multiple mediation model to investigate how occupational burnout influences the use of short-form video apps, which, in turn, enhances a sense of community and intrinsic rewards. Additionally, it explored how these factors improve life satisfaction among Chinese healthcare workers (aged 18–34 years old).

In terms of direct effects, as suggested by previous studies, frequent use of emerging social media is triggered when individuals experience persistent occupational burnout [42,43]. The results of the current study corroborate the previous literature and demonstrate that Chinese healthcare workers’ occupational burnout positively influences their use of short-form video apps. This also suggests that, similar to other demographic groups’ stress coping strategies [42,43,44,45], healthcare workers tend to choose digital leisure activities, such as engaging with short-form videos, to alleviate stress during periods of burnout. Therefore, this study enhances our understanding of how frontline workers in China use short-form video apps as a coping mechanism for occupational burnout.

Considering the indirect effect findings, the current study contributes to understanding the mediated mechanism of a sense of community in the relationship between the use of short-form video apps and life satisfaction among Chinese healthcare workers, a field that has not been fully investigated in workplace well-being in healthcare research. The results demonstrate that frequent use of short-form video apps enhances Chinese healthcare workers’ sense of community, which, in turn, reinforces their overall life satisfaction. These findings align with prior research, which suggests that frequent use of platforms such as Facebook can increase individuals’ perceived sense of community, thereby enhancing their life satisfaction [47,48]. Furthermore, the current study found that intrinsic rewards mediated the relationship between the use of short-form video apps and life satisfaction among Chinese healthcare workers. This finding is consistent with the theoretical framework that posits that increased group involvement enhances individuals’ sense of belonging and satisfies their needs. Additionally, the empirical outcome is consistent with earlier research showing that intrinsic rewards mediate the relationship between social network use and life satisfaction [59,60,61,62].

Unanticipated results were observed in testing the serial mediation hypotheses. Specifically, the use of short-form video apps and a sense of community did not exhibit a serial mediated effect in the relationship between occupational burnout and life satisfaction among Chinese healthcare workers. This finding contrasts with previous empirical studies in the fields of psychology and media use behavior [47,48,88]. One plausible explanation is that short-form video apps may encourage users to engage with content more mindfully rather than passively or mindlessly. This concept is known as the Joy of Missing Out (JOMO). Prior research has indicated that JOMO serves as a key antecedent to increased happiness [89]. Similarly, Nuket et al. (2022) found that, compared to mindless social media use (associated with the Fear of Missing Out, or FOMO), JOMO has a more significant positive impact on well-being [90]. Based on these findings, future research should explore whether a serial mediation mechanism exists between occupational burnout, the use of short-form video apps, JOMO, and life satisfaction (occupational burnout → the use of short-form video apps → JOMO → life satisfaction). Furthermore, the use of short-form video apps and intrinsic rewards did not show a serial mediation effect in the relationship between occupational burnout and life satisfaction among Chinese healthcare workers. These results contrast with previous findings [44,59,62]. One possible explanation for these differing results is that the current study defined healthcare workers as using a variety of short-form video apps to create, upload, and engage with a wide range of content, including liking, commenting, sharing, and socializing with other users. This includes both active and passive engagement with the apps. Growing scientific evidence suggests that different patterns of social media use can provide emotional relief, potentially leading to improved life satisfaction [91]. A prior study also emphasized the importance of distinguishing social networking site (SNS) use into active and passive categories and further investigating how each type affects individuals’ life satisfaction [92]. Supporting this view, another study found that active use of SNSs was a stronger predictor of increased life satisfaction [93]. In contrast, passive use of SNSs has been linked to lower well-being, mainly due to increased envy [87] and social comparison [94]. As previously discussed, various factors may have different impacts on Chinese healthcare workers.

However, contextual factors are particularly influential in shaping these effects. For instance, digital inclusion extends beyond simple internet access to include digital literacy and the effective use of essential digital platforms [95]. Research has identified digital inclusion as a key antecedent influencing individuals’ intentions to adopt information and communication technologies [96]. Additionally, healthcare workers demonstrate varying levels of digital proficiency, technological readiness, and access to investment in digital infrastructure [97]. These differences may influence the assumed serial mediation pathways in the relationship between occupational stress and social media use. Specifically, digital inclusion may moderate the strength of this relationship, meaning its impact varies depending on Chinese healthcare workers’ digital access and skills. Based on these findings, this study highlights the need for further research to explore the nuanced effects of digital inclusion among Chinese healthcare workers.

In terms of theoretical contributions, this study makes three key advances beyond prior knowledge. First, the theoretical framework of sense of community has predominantly been tested in Western contexts, while collectivistic cultural settings, particularly in healthcare and among frontline workers, have been less explored in relation to life satisfaction. Therefore, the current study expands the theoretical understanding of how the use of short-form video apps contributes to life satisfaction through a sense of community. Secondly, while many studies have already examined the impact of social media use on individuals’ life satisfaction within established theoretical frameworks, no study has yet explored how the use of emerging media, such as short-form video apps, enhances life satisfaction through external connections. This study is one of the first to confirm that occupational burnout positively influences Chinese healthcare workers’ use of short-form video apps. Lastly, a sense of community was found to be a critical mediator in the relationship between short-form video app use and life satisfaction. Based on these empirical findings, future research is encouraged to continue exploring the theoretical framework of a sense of community in various healthcare workplace and well-being contexts.

The findings of this study provide practical implications for developing campaigns in healthcare workplace and well-being contexts. Specifically, this study found that the use of short-form video apps positively influenced healthcare workers’ life satisfaction through two key mediators. However, prior research has highlighted that mindless social media use can lead to negative outcomes, such as increased envy [87] and social comparison [94]. To address these challenges, previous studies have emphasized the importance of using educational videos to enhance digital literacy [98]. Building on this insight, developing digital wellness educational content aimed at promoting healthy screen habits could help healthcare workers adopt mindful and intentional use of short-form video apps, thereby maximizing their positive effects on well-being. Furthermore, a recent report indicated that one of the most popular types of short-form video content among Chinese youth involves relaxation and training activities, such as yoga practices and sleep meditation [99]. These videos have garnered millions of likes and shares, highlighting substantial public interest and consistent growth in engagement. Scholarly evidence further supports that exposure to such relevant online content and participation in stress-relief activities positively impact users’ well-being and sleep quality [100,101]. Consequently, health departments and facilities should actively encourage healthcare workers to engage with these videos and incorporate the recommended activities into their routines to enhance overall well-being.

Despite the valuable insights provided by this study, several limitations should be acknowledged to guide future research. First, this study did not consider the potential influence of contextual factors, particularly the social environment. For example, subjective norms, defined as a person’s perception of social expectations to behave in a certain way, are an important social factor [102]. Subjective norms can be further categorized into injunctive norms (perceived expectations) and descriptive norms (observed behaviors) [103]. In the context of China, short-form video users often engage in stress relief activities inspired by descriptive norms, such as wellness habits showcased by recommended content creators or popular trends. This increased engagement highlights the importance of exploring the nuanced relationship between short-form video app use and life satisfaction via descriptive norms among healthcare workers in future studies. Additionally, the findings demonstrated a direct positive association between occupational burnout and healthcare workers’ use of short-form video apps. However, the statistical model accounted for only 11% of the variance (R^2^ = 0.11, *p* < 0.05), which warrants cautious interpretation. Lastly, a prior study stated that survey methods may overemphasize the limitations associated with respondent bias due to self-reporting of past behavior [104]. Similarly, this issue has been observed in the recall accuracy of respondents regarding their use of short-form video apps in the current study. Future research should consider designing more precise and reliable measurements for short-form video app usage to enhance the accuracy of findings and provide a deeper understanding of its effects on healthcare workers’ well-being.

## 6. Conclusions

Guided by the theoretical framework of sense of community, the current study investigated a serial multiple mediation model to explore how occupational burnout is positively associated with the use of short-form video apps, which, in turn, enhances a sense of community and intrinsic rewards. Moreover, how these factors contribute to enhancing life satisfaction among Chinese healthcare workers aged 18–34 years was investigated. Using carefully designed measures and a sample (N = 362) collected via an online questionnaire, the study sequentially tested direct, mediated, and serially mediated hypotheses using SPSS 25.0. The findings provide valuable insights and practical implications, offering potential strategies to enhance well-being in healthcare settings. Specifically, developing digital wellness educational content and encouraging healthcare workers to engage with relaxation and training videos could maximize the positive effects of short-form video usage on well-being. Future research is encouraged to further explore the nuanced effects of serial mediation, particularly in relation to the concepts of JOMO and digital inclusion.

## Figures and Tables

**Table 1:** Fornell–Larcker criterion for discriminant validity.

Variables	1	2	3	4	5
Occupational burnout	0.87				
USFVA	0.10 *	0.89			
Sense of community	0.18 **	0.44 **	0.86		
Intrinsic rewards	0.18 **	0.26 **	0.58 **	0.86	
Life satisfaction	−0.08	0.44 **	0.45 **	0.37 **	0.89

* *p* < 0.05, ** *p* < 0.01.

**Table 2:** Demographic characteristics of survey participants.

Variables	Item	Number	Percentage
Sex	Female	165	45.6%
	Male	197	54.4%
Education level	Pre-college	36	10.0%
	Undergraduate	285	78.7%
	Postgraduate	41	11.3%
Age	18–23 years old	202	55.8%
	24–29 years old	63	17.4%
	30–34 years old	97	26.8%
Monthly household income (CNY)	3000–5000	86	23.7%
	5001–8000	89	24.6%
	8001–15,000	119	32.9%
	15,001 and higher < CNY	68	18.8%
Type of work	Medical intern	201	55.5%
	Physician and surgeon	41	11.3%
	Pharmacist	26	7.2%
	Nurse	50	13.8%
	Aide	44	12.2%
Total		362	100%

**Table 3:** Summary of hypothesis testing results.

Hypotheses	Relationship	Result
Hypothesis 1	Occupational burnout has a significant and positive effect on healthcare workers’ USFVA.	Supported
Hypothesis 2	Sense of community mediates the relationship between USFVA and Chinese healthcare workers’ life satisfaction.	Supported
Hypothesis3	Intrinsic rewards mediate the relationship between USFVA and Chinese healthcare workers’ life satisfaction.	Supported
Hypothesis 4	USFVA and a sense of community play a serial mediating role in the relationship between occupational burnout and Chinese healthcare workers’ life satisfaction.	Rejected
Hypothesis 5	USFVA and intrinsic rewards play a serial mediating role in the relationship between occupational burnout and Chinese healthcare workers’ life satisfaction.	Rejected

## Data Availability

The original data are provided by all the authors. If there are relevant research needs, the data can be obtained by sending an email to the corresponding author. Please indicate the purpose of the research and a statement of data confidentiality in the email.

## Appendix A

**Table A1:** Measurement items and model assessment metrics.

Variables	Source	Items	Loading	α	CR	AVE
Occupational Burnout	[71,72]	I feel overwhelmed by my workload.	0.84	0.87	0.97	0.73
		My work negatively impacts my physical health (e.g., causing illness).	0.88			
		My job leaves me with very limited time to complete my tasks at work.	0.88			
		My job leaves me with very limited time for personal or family activities.	0.83			
USFVA	[73,74]	Liking short-form video content that I enjoy.	0.86	0.86	0.97	0.70
		Leaving comments in the comment section.	0.83			
		Favoriting short-form video content that I like.	0.83			
		Sharing short-form video content with family and friends.	0.80			
Sense of Community	[75,76]	I get my important needs fulfilled by being part of the community of short-form video apps	0.88	0.86	0.97	0.78
		When I have a problem, I can discuss it with other users in the community of short-form video apps.	0.89			
		Fitting into the community of short-form video apps is important to me.	0.89			
Intrinsic Rewards	Newly created	When using short-form video apps, I often feel relaxed and relieved from stress.	0.89	0.89	0.98	0.75
		When using short-form video apps, I experience a sense of fulfillment through gaining knowledge or enhancing my skills	0.92			
		Short-form video apps enable me to enjoy the process of exploring new knowledge.	0.91			
		Short-form video apps provide me with a sense of emotional resonance and joy.	0.75			
Life Satisfaction	[77,78]	Overall, I am satisfied with my life.	0.89	0.93	0.98	0.79
		I feel satisfied in my relationships with others.	0.86			
		When people say they are satisfied with life, I often share the same feeling.	0.90			
		The conditions of my life are excellent.	0.93			
		I have a positive outlook on my future.	0.86			
